# Gestational intermittent hypoxia increases FosB‐immunoreactive perikaryas in the paraventricular nucleus of the hypothalamus of adult male (but not female) rats

**DOI:** 10.1113/EP091343

**Published:** 2023-08-29

**Authors:** Danuzia Ambrozio‐Marques, Marianne Gagnon, Abigail B. Radcliff, Armand L. Meza, Tracy L. Baker, Jyoti J. Watters, Richard Kinkead

**Affiliations:** ^1^ Research Center of the Québec Heart and Lung Institute Université Laval Quebec City Québec Canada; ^2^ Department of Comparative Biosciences, School of Veterinary Medicine University of Wisconsin Madison Wisconsin USA

**Keywords:** cardiorespiratory control, development, intermittent hypoxia, stress

## Abstract

Sleep‐disordered breathing is a respiratory disorder commonly experienced by pregnant women. The recurrent hypoxaemic events associated with sleep‐disordered breathing have deleterious consequences for the mother and fetus. Adult male (but not female) rats born to dams subjected to gestational intermittent hypoxia (GIH) have a higher resting blood pressure than control animals and show behavioural/neurodevelopmental disorders. The origin of this persistent, sex‐specific effect of GIH in offspring is unknown, but disruption of the neuroendocrine stress pathways is a key mechanism by which gestational stress increases disease risk in progeny. Using FosB immunolabelling as a chronic marker of neuronal activation, we determined whether GIH augments basal expression of FosB in the perikaryas of cells in the paraventricular nucleus of the hypothalamus (PVN), a key structure in the regulation of the stress response and blood pressure. From gestational day 10, female rats were subjected to GIH for 8 h/day (light phase) until the day before delivery (gestational day 21); GIH consisted of 2 min hypoxic bouts (10.5% O_2_) alternating with normoxia. Control rats were exposed to intermittent normoxia over the same period (GNX). At adulthood (10–15 weeks), the brains of male and female rats were harvested for FosB immunohistochemistry. In males, GIH augmented PVN FosB labelling density by 30%. Conversely, PVN FosB density in GIH females was 28% lower than that of GNX females. We conclude that GIH has persistent and sex‐specific impacts on the development of stress pathways, thereby offering a plausible mechanism by which GIH can disturb neural development and blood pressure homeostasis in adulthood.

## Abstract (highlights)

- **What is the central question of this study?**
  In pregnant women, sleep apnoea increases the risk of disease for the offspring at various life stages. Given that gestational stress disrupts the programming of the stress pathways, we determined whether exposing female rats to gestational intermittent hypoxia (GIH) activates hypothalamic neurons regulating the stress response in adult rats.
- **What is the main finding and its importance?**
  Using FosB immunolabelling as a marker of marker of neuronal activation, we showed that GIH augmented basal activation of the paraventricular nucleus of the hypothalamus in males, but not females. Disruption of the stress pathways is a new hypothesis to explain the persistent and sex‐specific impacts of GIH on offspring health.

## 1 INTRODUCTION

Sleep‐disordered breathing (SDB) is a complex, multifactorial respiratory disorder that affects nearly 1 billion adults worldwide (Benjafield et al., 2019). It is characterized by repeated episodes of respiratory arrest, with or without upper airway obstruction, and these apnoeic events often result in hypoxaemia, hypercapnia and sleep fragmentation. Those consequences of apnoeas are powerful stressors causing significant endocrine and metabolic disturbances (Cho et al., 2019; Conde et al., 2023; Martins & Conde, 2021; Prabhakar et al., 2012).

The clinical manifestations and prevalence of SDB are sex specific (Kinkead et al., 2021). The occurrence of SDB is ∼2.5 times higher in men than in premenopausal women (Heinzer et al., 2015; Peppard et al., 2013), but pregnancy increases the occurrence of SDB from 10% to 27% between the first and third trimester, thus reaching a level similar to men (Dominguez et al., 2018; Pien et al., 2014; Young et al., 1993). When experienced during gestation, SDB increases the risk of cardiovascular disease, diabetes and mood disorders for the mother (Dominguez & Habib, 2022) and augments the incidence of preterm birth and low birth weight (Chen et al., 2012; Reyes‐Zúñiga & Torre‐Bouscoulet, 2016). Although the direct impacts of gestational SDB on the developmental trajectories of infants are difficult to document, we know that adult male (but not female) rats born to dams subjected to gestational intermittent hypoxia (GIH) have higher resting blood pressure (Song et al., 2022). Additionally, GIH males show significant neurodevelopmental and behavioural alterations that are similar to autism spectrum disorder in humans (Vanderplow et al., 2022).

The mechanisms by which GIH has persistent and sex‐specific impacts on cardiovascular homeostasis and the behaviour of the offspring are unknown, but the phenotypic similarities between GIH‐subjected rats and that of animals that experienced stress during early life raise the possibility that dysfunction of the stress pathways is at the core of the problem (Tenorio‐Lopes & Kinkead, 2021). In mammals (including humans), exposure to maternal separation during early life disrupts the programming of the hypothalamo–pituitary–adrenal (HPA) axis and increases basal activation of the circuits and responsiveness to stress in adulthood, especially in males (Tenorio‐Lopes & Kinkead, 2021). This condition augments the risk for a broad range of diseases, including diabetes, mental health issues and cardiovascular diseases (Lombard, 2010; McEwen & Gianaros, 2010; Pietrobon et al., 2020; Shonkoff et al., 2009). This explanation is attractive but requires testing, because the effects of stress on physiological systems and behaviours depend on the nature of the challenge, the timing and the duration of exposure. Gestational intermittent hypoxia is a systemic stressor that, unlike psychological stress, poses an immediate and direct threat to the physiological health of the mother and the fetus. Furthermore, it has been reported that exposure of pups to intermittent hypoxia (IH) during the neonatal period has limited influence on HPA function (Chintamaneni et al., 2013).

As a proof of concept, the present study evaluated the impacts of GIH on the basal function of the HPA axis in adult male and female rats. We used immunohistochemistry to compare the expression of the transcription factor FosB, a chronic marker of neuronal activation (Perrotti et al., 2004), between rats born to GIH‐exposed dams and control animals. Our analyses focused on the paraventricular nucleus of the hypothalamus (PVN), a key structure in the regulation of the HPA axis and blood pressure (Dampney et al., 2018; Ulrich‐Lai & Herman, 2009). We also quantified FosB expression in the amygdala, because changes in local regulation of excitability of the basolateral subregion underlie behavioural disturbances characteristic of disorders including post‐traumatic stress syndrome, autism and attention‐deficit hyperactivity disorder (Sharp, 2017).

## 2 MATERIALS AND METHODS

### 2.1 Ethical approval

All protocols and practices were performed according to the ARRIVE (2.0) and US National Institutes of Health guidelines set forth in the *Guide for the Care and Use of Laboratory Animals* and with protocols approved by the University of Wisconsin–Madison Institutional Animal Care and Use Committee (protocol V005173‐R02).

### 2.2 Animals and GIH protocol

Pregnant Sprague–Dawley rats at gestational day (G) 9 were obtained from Charles River (Wilmington, MA, USA) and housed in AAALAC‐accredited facilities with food and water ad libitum and 12 h–12 h light–dark conditions. Intermittent hypoxia was used to simulate the oxygen desaturation and reoxygenation that occurs in pregnant women with SDB. Based on our previous protocol (Vanderplow et al., 2022), GIH began at G10 and was terminated at G21, before delivery. During that time, pregnant females were placed in cages with custom‐made Plexiglass lids for controlled exposure to hypoxic or normoxic conditions for 8 h/day (from 09.00 to 17.00 h). Intermittent hypoxia consisted of 2 min hypoxic bouts (45 s down to 10.5% O_2_) alternating with 2 min of normoxia (15 s up to 21% O_2_). Preceding delivery (G22), the custom‐made Plexiglass lids were replaced with standard filter toplids to avoid direct exposure of the offspring to IH. Control rats were exposed in parallel to ensure that all groups were exposed to the same experimental conditions; however, control rats received only alternating episodes of room air (normoxia; 21% O_2_; group GNX), with the same gas flow used for the IH protocol. After birth, GNX and GIH pups were maintained in standard housing conditions in our animal care facilities. At postnatal day (P) 21, juvenile animals were weaned and housed in pairs until their brains were collected. Each group was composed of five or six animals (females and males; GNX and GIH); individual data points in the figures indicate the specific number of replicates in each group. Each group was composed of animals from three litters, apart from the GIH females, which came from two litters.

### 2.3 Immunohistochemical staining for FosB

At 10–15 weeks of age, male and female rats were killed by transcardiac perfusion. Rats were first deeply anaesthetized with 5% isoflurane in air via the drop method. Once the animal lost the pedal withdrawal response to pinch, the rat was perfused intracardially with 1× PBS (1 mL/g body weight, pH 7.4), followed by 4% paraformaldehyde (1 mL/g body weight) diluted in 1× PBS. Brains were dissected, post‐fixed with 4% paraformaldehyde at 4°C for 48 h, then immersed in 30% sucrose solution for 48 h at 4°C. Finally, brains were frozen in dry ice and kept at −80°C until they were sectioned.

Frozen brains were cut with a sliding microtome at 40 μm using the ‘dry ice’ method (Anders, 1942). Coronal sections were placed in a cold cryoprotectant solution (0.05 M sodium phosphate buffer, 30% ethylene glycol and 20% glycerol) and stored at −20°C. Finally, FosB immunolabelling was performed according to standard procedures (Ansorg et al., 2015).

During all steps, plates holding tissue sections were placed on a shaker (VEVOR adjustable variable speed oscillator orbital rotator shaker, CA, USA) at 20 r.p.m. Free‐floating tissue sections were washed with Tris‐buffered saline (TBS) pH 7.4 for 1 min at room temperature. Then, heat‐induced antigen retrieval was performed by incubating sections in a solution of 0.1 M citric acid and 0.1 M sodium citrate diluted in water (pH 6.0) for 5 min at 65°C, and 10 min at 95°C (Yamashita & Katsumata, 2017). Tissue sections were then rested for 30 min until they reached room temperature. Next, sections were washed using TBS (three times, each for 5 min) and, sequentially, had the endogenous peroxidase activity blocked using 3% H_2_O_2_ in TBS for 30 min, followed by a wash in TBS (three times, each for 5 min). Sections were then placed in a blocking solution (1% BSA and 0.4% Triton X‐100 in TBS) for 1 h to reduce non‐specific binding. Next, sections were incubated with the primary FosB antibody (#2251; Cell Signaling Technology, MA, USA) made in rabbits (dilution 1:2000 in the blocking solution) at 4°C overnight.

The next day, sections were placed at room temperature for 1 h before being washed three times in TBS (for 5 min each), and incubated with a biotinylated secondary antibody for 3 h at room temperature (goat anti‐rabbit IgG, 1:400 dilution; Vector Laboratories; diluted in blocking solution). Sections were then incubated in the Vectastain Elite avidin–biotinylated enzyme complex (ABC) (Vector Laboratories, CA, USA) for 90 min (Ansorg et al., 2015). After ABC incubation, a wash was performed in TBS (three times, each for 5 min), and the nickel chloride diaminobenzidine peroxidase method (SigmaFast DAB with metal enhancer; Sigma Aldrich, St Louis, MO, USA) was used; incubating the sections for 4 min revealed the biotinylated secondary antibody. A final wash was performed using TBS (three times, each for 5 min). Finally, the sections were mounted on Fisherbrand tissue path superfrost plus gold slides (Thermo Fisher Scientific, MA, USA) and left to dry for 48 h, after which they were coverslipped with permanent mounting medium to be visualized under the microscope.

### 2.4 Identification of regions of interest and quantification of FosB‐immunoreactive cells

**FIGURE 1:**
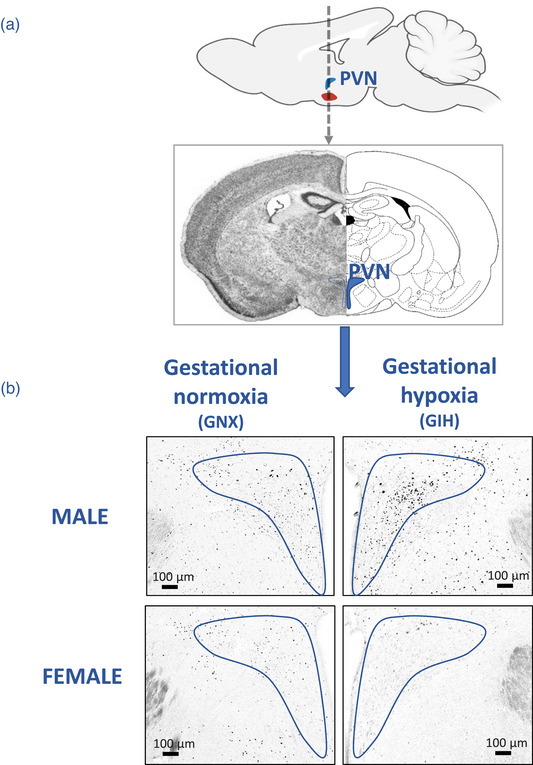
(a) Top: Sagittal view of the rat brain, illustrating the location of the paraventricular nucleus of the hypothalamus (PVN). The dashed arrow indicates the location of the tissue sections and points to a coronal view of the area of interest (bottom panel). (b) Representative photomicrographs comparing FosB immunolabelling in the PVN of adult rats born to females exposed to gestational normoxia (GNX; left) with that of rats exposed to gestational intermittent hypoxia (GIH; right). Data are reported for males (top) and females (bottom).

**FIGURE 2:**
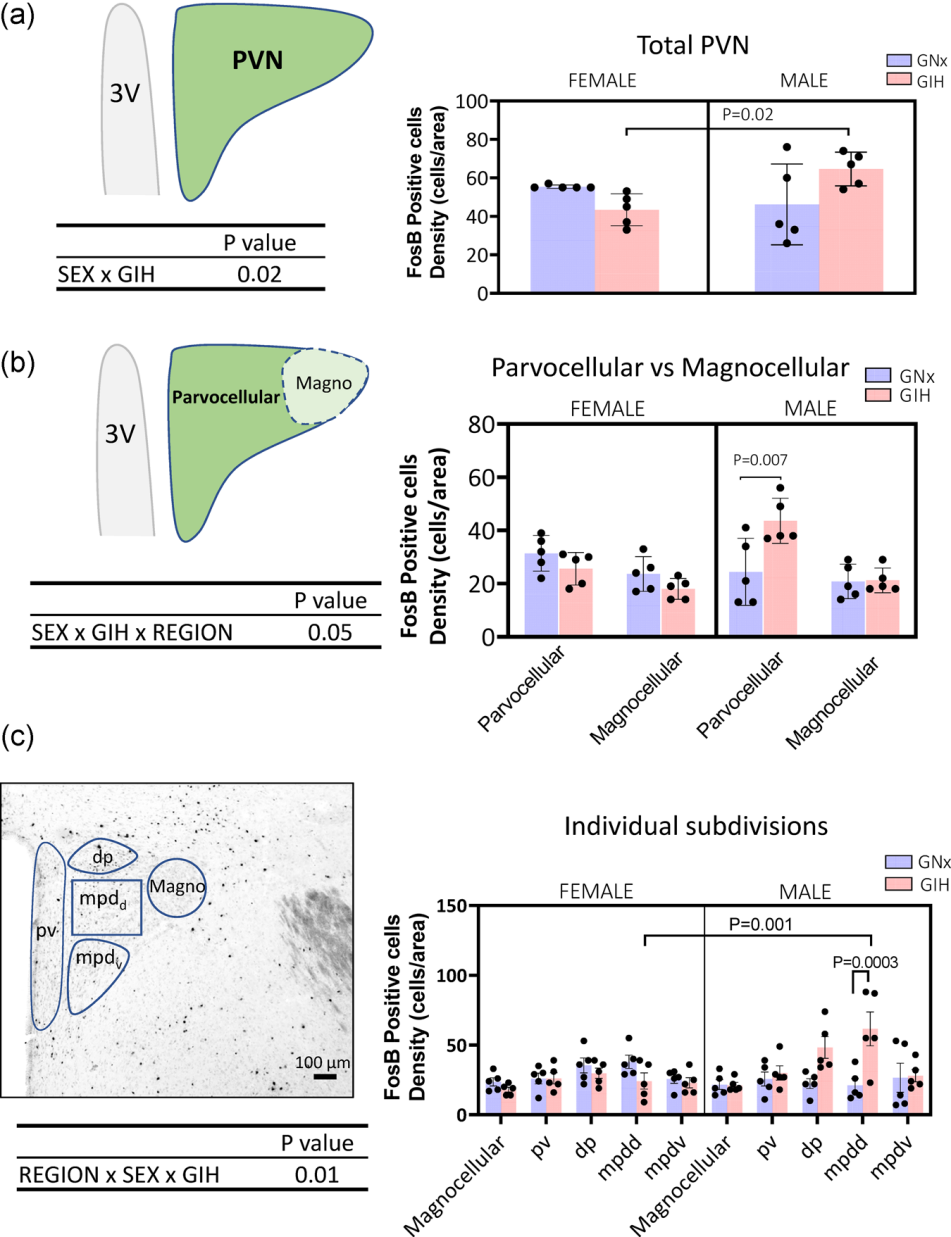
(a) Left: Schematic representation of the paraventricular nucleus of the hypothalamus (PVN), showing where the density of FosB‐positive cells was counted. Right: Bar graphs presenting the individual data for each group. The bar height indicates the group mean ± SD. The ANOVA result is reported below the schematic diagram. (b) Left: Schematic representation of the two main subdivisions of the PVN where the density of FosB‐positive cells was counted. Right: Bar graphs presenting the individual data for each group and area. The bar height indicates the group mean ± SD. The ANOVA result is reported below the schematic diagam. (c) Left: Photomicrograph of the PVN, illustrating regions of interest where the density of FosB‐expressing cells was quantified. Specifically, these include the magnocellular (Magno) area and the four main subdivisions of the parvocellualr region of the PVN. Right: Bar graphs presenting the individual data for each group and area. The bar height indicates the group mean ± SD. The ANOVA results are reported below the schematic diagram. The *P*‐values of *post hoc* tests are indicated above the brackets above the means being compared. Abbreviation: 3V, third ventricle.

**FIGURE 3:**
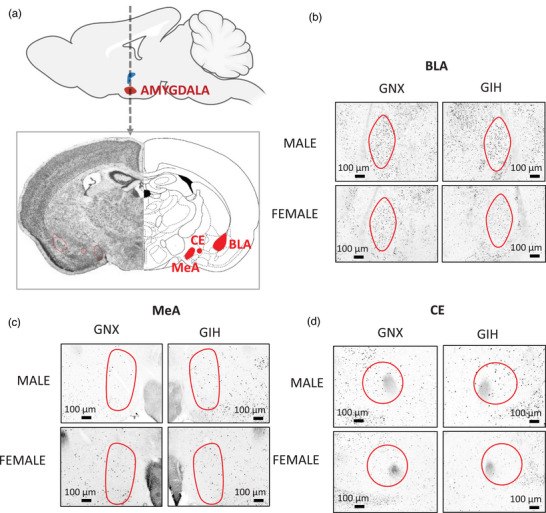
(a) Top: Sagittal view of the rat brain, illustrating the location of the amygdalar complex. The dashed arrow indicates the location of the tissue sections, and the bottom panel shows a coronal view of the amygdala and its main subdivisions: central (CeA), basolateral (BLA) and medial (MeA) areas. (b–d) Representative photomicrographs comparing FosB immunolabelling in the BLA (b), MeA (c) and CE (d) of adult rats born to females exposed to gestational normoxia (GNX; left) with that of rats exposed to gestational intermittent hypoxia (GIH; right). Data are reported for males (top) and females (bottom).

Slides with the FosB‐immunolabelled tissue were visualized under a microscope (Eclipse E600; Nikon, Tokyo, Japan) equipped with a camera (Infinity 3; Lumenera Corporation, ON, Canada) and stored as digital images. Images used for data analysis were captured with a ×10 magnification objective. FosB protein expression was used as a functional marker of chronic neuronal activation (Nestler, 2012); analyses were performed in the PVN (bregma: −1.80 to −1.88 mm) and the amygdalar complex (bregma: −2.56 to −2.80 mm) based on illustrations from the rat brain stereotaxic atlas (Paxinos & Watson, 1998). The shape of the third ventricle and the white matter of the optic tract were used as anatomical landmarks (see Figures 1, 2 and 3a).

The PVN is a complex structure composed of diverse neuronal phenotypes that contribute to the regulation of motivated behaviours, neuroendocrine and autonomic responses to stress (Sladek et al., 2015). To obtain more precise insight into the effects of GIH on the PVN and potential physiological repercussions, our analysis initially considered the two major areas of the PVN (Figures 2b,c) based on their distinct anatomical characteristics and functional contributions. Magnocellular neurons are large (13−19 μm) neurosecretory cells that project to the posterior pituitary, including neurosecretory cells that release vasopressin, which influences blood pressure. Parvocellular neurons are small neurons (6−10 μm) with distinct phenotypes and projections (Kiss et al., 1991); here, we considered the dorsal and ventral subdivisions of the dorsal medial parvocellular (mpd_d_ and mpd_v_, respectively) and the periventricular region (pv). Of note, the mpd receives noradrenergic projections from the nucleus of the solitary tract (Cunningham & Sawchenko, 1988), the primary projection site of the carotid bodies (Housley et al., 1987). Finally, these three subregions project to the median eminence, where they secrete numerous hormones and neurotransmitters; their influence on blood pressure is via pre‐autonomic actions (Sladek et al., 2015). Furthermore, the dorsal subdivision (dp) also exerts a pre‐autonomic influence owing to its projections to the brainstem and spinal cord (Sladek et al., 2015).

The amygdalar complex is also composed of distinct structures associated with specific neurological functions; however, all three structures contribute to anxiety‐related behaviours (Dbiec & Ledoux, 2009; Kinkead et al., 2023; Zhang et al., 2021). Thus, our analysis considered the basolateral (BLA), central (CeA) and medial (MeA) subdivisions of the amygdala (Dbiec & Ledoux, 2009; Zhang et al., 2021; see Figure 3). Briefly, the BLA is of interest because this subregion communicates bi‐directionally with brain regions affecting cognition, motivation and stress responses; enhanced excitability within this structure underlies behavioural disturbances characteristic of neurodevelopmental disorders such as autism and attention‐deficit hyperactivity disorder (Sharp, 2017), and such traits have been reported in GIH‐exposed male offspring (Vanderplow et al., 2022). From a respiratory perspective, the BLA contains CO_2_‐sensing neurons capable of eliciting fear‐related behaviour (Ziemann et al., 2009). Of note, the MeA shows sex‐based differences in anatomy, laterality, function and sensitivity to steroid hormones (Buss et al., 2012; Rodrigues et al., 2009). Both regions project to the CeA (Keshavarzi et al., 2014), which is the output pathway of the amygdala because it projects directly onto rhythmogenic neurons of the pre‐Bötzinger complex, the nucleus of the solitary tract and the retrotrapezoid nucleus (Petrov et al., 1995; Rosin et al., 2006; Ulrich‐Lai & Herman, 2009; Yang et al., 2020).

The analyses were conducted in a randomized order, and the experimenters were blinded to the identities of the animals and treatments. The representative photomicrographs reported in Figures 1b and 3b–d show that the clear contrast of the FosB‐labelled nucleus facilitated quantification. To facilitate comparisons between areas of different sizes, the number of immunopositive cells was expressed as a function of the size of the structure (cell density) using standardized templates that were built according to the specific form of the structure (see schematic representations in Figures 2a and 3a). For each section, the right and left sides were quantified and the results averaged; ANOVA did not reveal any evidence of lateralization. The templates of the structures (Figures 1b and 3) were applied onto the pictures using ImageJ (NIH software; US National Institutes of Health System), and the quantification was performed manually. The specific areas of each structure were as follows: parvocellular, 0.51 × 10^5^ μm^2^; pv, 0.36 × 10^5^ μm^2^; dp, 0.21 × 10^5^ μm^2^; mpdd, 0.51 × 10^5^ μm^2^; mpdv, 0.44 × 10^5^ μm^2^; BLA, 1.79 × 10^5^ μm^2^; CE, 1.49 × 10^5^ μm^2^; and MeA, 1.45 × 10^5^ μm^2^.

### 2.5 Statistical analyses

**FIGURE 4:**
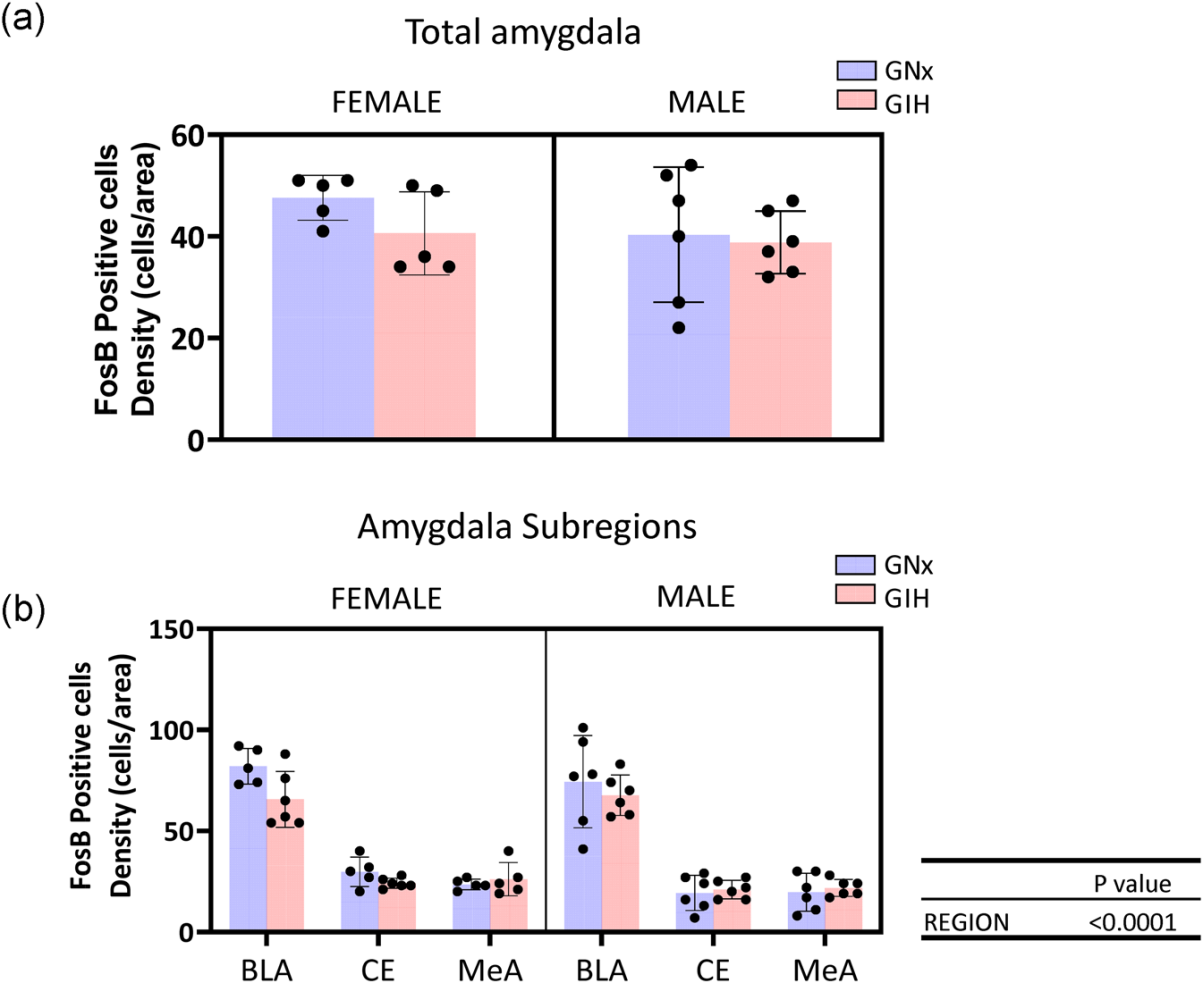
(a) Bar graphs presenting idividual data and comparing the density of FosB‐expressing perikayas in the amygdala of rats born to dams subjected to intermittent normoxia (GNX; blue) or intermittent hypoxia (GIH, red); data are reported for females and males (left and right, panels, respectively). (b) A more refined analysis considering the three areas of the amygdala [basolateral (BLA), central (CE) and medial (MeA)]. The bar height indicates the group mean ± SD.

Statistical analyses were performed using ANOVA. For the results reported in Figure 2a, we first analysed the entire PVN using a two‐way ANOVA, considering sex and treatment as factors. Region‐specific effects of GIH were then assessed using a three‐way ANOVA that added the region as a factor (Figure 2b,c). A similar approach was used for analysis of the amygdala (Figure 4). When ANOVA results were significant, the analysis was followed by the Holm–Sidak *post hoc* test to identify specific group differences. The statistical analyses were performed using GraphPad Prism software (v.8.4.2 for Windows), and the same software was used to plot the figures. For clarity of the text, ANOVA results are reported in the figures. The significance level was set to *P* < 0.05. Results from *post hoc* tests are reported in the figures by reporting their *P*‐values. The figures report individual data, and the height of the bars indicates the group mean ± SD.

## 3 RESULTS

### 3.1 Sex‐ and region‐specific effects of GIH on the density of FosB‐immunoreactive cells

#### 3.1.1 Immunopositive cells in the PVN

The representative photomicrographs of FosB immunolabelling (Figure 1b) and the population data reported in Figure 2a illustrate the overall sex‐specific effects of GIH on the density of FosB‐positive cells in the entire PVN. The mean density of labelled cells observed in females exposed to GIH was 28% lower than that for GNX; conversely, in GIH males FosB labelling density was augmented by 30%. Although the ANOVA supports a sex‐specific effect, *post hoc* tests were not significant owing to the low number of replicates. However, a more detailed analysis of the parvo‐ and magnocellular areas showed that the largest increase in FosB after GIH exposure was in the parvocellular area of males (79% higher). This effect was not observed in females. Subsequent analysis of the parvocellular subdivisions (Figure 2c) showed that the largest FosB responses to GIH were in the mpdd subdivision (190% increase). Again, those region‐specific effects were not observed in females.

#### 3.1.2 Immunopositive cells in the amygdala

Analysis of the entire amygdalar complex showed that neither sex nor treatment influenced the density of FosB labelling (Figure 4a). A more detailed anatomical consideration showed that the highest density of FosB‐positive cells was in the BLA; however, neither sex nor GIH affected expression in any of the subregions considered (Figure 4b).

## 4 DISCUSSION

The fetal stage is a critical period of development, and the impacts of adverse events on the offspring depend greatly on the nature, intensity and timing of the stress. Preclinical studies demonstrate that at adulthood, male (but not female) rats born to dams subjected to GIH are at risk for hypertension (Song et al., 2022) and show abnormal neurodevelopmental traits associated with autism spectrum disorder (Vanderplow et al., 2022). Given that abnormal programming of the neural circuits regulating the stress pathways is a key mechanism by which stress experienced in utero contributes to the emergence of cardiovascular disease, diabetes and behavioural disorders later in life (Hodes & Epperson, 2019; Kapoor et al., 2006), we hypothesized that GIH results in a sex‐specific increase of basal HPA axis activation in adult rats. Quantification of FosB expression in the PVN provides solid support for our hypothesis. In an attempt to explain why GIH induces behavioural manifestations of abnormal neurodevelopmental disorders in males (Vanderplow et al., 2022), we also quantified FosB expression in the amygdala, but the absence of an effect within this structure indicates that this structure was not activated by GIH.

### 4.1 How does GIH affect the fetus?

Maternal stress leads to numerous cardiovascular and endocrine responses in the mother, including the release of ACTH, glucocorticoids and catecholamines into the bloodstream. These stress signals can affect the fetus (Yang, 1997), but the placenta forms a structural and biochemical barrier that attenuates the levels reaching the fetus; however, the ‘protection’ offered by the placenta has its limits. When sufficiently severe, stress can activate the fetal HPA axis via direct or indirect mechanisms (Challis et al., 2000; Kapoor et al., 2006). For instance, secretion of catecholamines by the mother can reduce placental blood flow and lead to placental hypoxia (Kapoor et al., 2006); this, in turn, can activate the HPA axis (and sympathetic system) of the fetus (Challis et al., 2000). Given that the fetus is protected from low O_2_ by the binding characteristics of fetal haemoglobin, a direct action of hypoxia on the fetus would require a very severe drop in O_2_. However, compensatory mechanisms activated by hypoxia, such as CO release and/or vascular remodelling, might contribute to the problem. Furthermore, it is also possible that excessive glucocorticoids might reach the fetus. Regardless, the lack of measurement of stress hormones in the mother and fetus during and/or at the end of the protocol prevents us from proposing a detailed explanation of the mechanism by which GIH leads to enhanced FosB labelling in the PVN of the offspring. This limitation will be addressed in subsequent studies dedicated to this question.

### 4.2 Can the increased FosB in the PVN explain the high blood pressure observed in GIH‐exposed rats?

The PVN receives multiple inputs, ranging from the forebrain to the hindbrain (Sladek et al., 2015). The PVN has three main effector pathways: neurosecretory magnocellular, neurosecretory parvocellular and pre‐autonomic parvocellular (Savić et al., 2022; Sladek et al., 2015). As already mentioned, GIH leads to a sex‐specific increase in blood pressure in adult male offspring (Song et al., 2022). Thus, depending on the region they activate, afferent signals can influence blood pressure by stimulating the endocrine and autonomic premotor responses of the PVN that will result in slow or rapid effects, respectively. The mpd_d_ subregion of the parvocellular PVN is composed almost exclusively of neurosecretory cells that release corticotrophin‐releasing hormone into the anterior pituitary (Sladek et al., 2015). Accordingly, GIH males are likely to have higher levels of circulating corticosterone at rest; however, this is yet to be demonstrated experimentally. This would be informative because with time, high levels of circulating corticosterone favour oxidative stress (Spiers et al., 2015), a condition that contributes to multiple health issues, including those induced by chronic IH (Kivimäki & Steptoe, 2018; McEwen, 2008).

In the present study, the fact that the effect of GIH on PVN activation was not observed in females is consistent with the sex‐specific effects of other forms of stress on the development of HPA function (Tenorio‐Lopes & Kinkead, 2021) and is in line with the cardiovascular profile of GIH‐exposed female offspring. In general terms, this sex‐based difference can be ascribed to the protective actions of oestrogens against the deleterious consequences of chronic stress on the HPA axis and cardiorespiratory function (Laouafa et al., 2019; Ribon‐Demars et al., 2019; Song et al., 2022; Tenorio‐Lopes & Kinkead, 2021).

### 4.3 Does GIH affect the amygdala?

Besides increasing the risk of cardiovascular disorders, gestational stress has been linked to neurobehavioural disorders, including attention‐deficit hyperactivity disorder, autism spectrum disorder and the incidence of anxiogenic and depressive‐like behaviour in rats and non‐human primates (Hodes & Epperson, 2019; Lautarescu et al., 2020; Van den Bergh et al., 2020).

The amygdala is listed among structures affected by gestational stress (Weinstock, 2005), and data from rodents and humans show that prenatal stress augments its responsiveness to subsequent stressors (Malter Cohen et al., 2013). Although the results reported here provide no evidence indicating that the basal level of activity of the amygdalar complex was affected by GIH, the impact of a subsequent challenge on the response of the amygdala (or any other outcome) was not tested. At this stage, our results do not preclude the potential impacts of GIH on behaviour.

## 5 CONCLUSION

By showing that GIH augments FosB labelling in the PVN of adult males, our study provides the first evidence that GIH leads to persistent and sex‐specific disruption of HPA function in offspring. This observation, along with the region‐specific effect of GIH on FosB labelling in the PVN, indicate that an increased pre‐autonomic influence on blood pressure is a plausible explanation for the hypertension reported in males born to GIH‐exposed dams. Although increased HPA function might contribute to other (behavioural) traits, further experiments exploring other protocols, such as responsiveness to a second stressor, are necessary to establish such links. Overall, our findings highlight the importance of gestational health leading to long‐term consequences for the offspring.

## AUTHOR CONTRIBUTIONS

D. Ambrozio‐Marques, M. Gagnon, A. Radcliff and A. Meza performed the experiments. D. Ambrozio‐Marques and M. Gagnon analysed the results and prepared the figures. All authors contributed to the writing of the manuscript, approved the final version of the manuscript and agree to be accountable for all aspects of the work in ensuring that questions related to the accuracy or integrity of any part of the work are appropriately investigated and resolved. All persons designated as authors qualify for authorship, and all those who qualify for authorship are listed.

## CONFLICT OF INTEREST

The authors declare no conflicts of interest.

## Data Availability

Original data are available upon request.
